# A transcription unit based systems biology study on *Salmonella* typhimurium gene organization, evolution, co-expression, and regulation

**DOI:** 10.3389/fmicb.2026.1816957

**Published:** 2026-05-21

**Authors:** Leting Sun, Zhixuan Deng, Junya Zhang, Xin Cao, Shuhong Liu, Runhong Chen, Min Zheng, Yejun Wang

**Affiliations:** 1 Youth Innovation Team of Medical Bioinformatics, Shenzhen University Medical School, Shenzhen, China; 2 Department of Cell Biology and Genetics, College of Basic Medicine, Shenzhen University Medical School, Shenzhen, China; 3 Undergraduate Innovation Platform, Experiment Center for Medical Teaching, Shenzhen University Medical School, Shenzhen, China

**Keywords:** core transcription unit, operon, operon evolution, pan transcription unit, *Salmonella* typhimurium, transcription unit

## Abstract

A bacterial transcription unit (TU) could be composed of a single gene or multiple adjacent genes forming an operon. Traditional systems biology often relies on gene-centric analysis, overlooking the regulatory complexity inherent in the operon structure, that is, containing transcriptional regulatory elements shared by multiple genes within a unique operon. Here, by integrating genome annotation with large-scale transcriptomic profiling data, we systematically identified operons from five representative *Salmonella* typhimurium strains, followed by comparative TU analyses among the strains. The core TUs are conserved among strains and govern essential metabolic functions. In contrast, the non-core pan TUs exhibit higher regulatory plasticity, driving environmental adaptation and strain-specific biological processes. Comparative TU analysis also reveals three main variation patterns of the pan-operon families across the strains, among which, alternative organization of operons with the same set of genes among strains happens frequently. We further decomposed the TUs into co-expression network modules and delineated the regulatory network for each module. Notably, we identified two modules, CEN9 and CEN5, which are enriched with genes of *Salmonella* Pathogenicity Island 1 (SPI-1) and SPI-2 type III secretion systems and the substrates, and therefore potentially related with bacterial virulence and systemic infection, respectively. In summary, the study proposed a new transcriptome data based TU-detecting strategy, based on which we identified the TUs in *S*. typhimurium, observed their evolutionary patterns, and comprehensively elucidated their organization and regulation.

## Introduction

In bacteria, functionally related genes are often organized into operons (Jacob and Monod, 1961). Genes within the same operon are located close to each other in the genome, often share the same transcriptional regulatory elements and form a unified transcription unit (TU; Browning and Busby, 2004; Rocha, 2008; Bundalovic-Torma et al., 2020; Del Duca et al., 2023). This organization allows bacteria to efficiently coordinate gene expression in response to varying environmental conditions (Osbourn and Field, 2009; Lim et al., 2011). However, not all composed by operons, bacterial TUs could also be formed by single genes, which are transcribed and transcriptionally regulated independently. In many bacterial systems biology studies, individual genes rather than TUs are considered as the fundamental analytic units. Despite valuable insights that have been derived from such gene-centric studies, it is inherently limited for the methodology which failed to capture the complexity of gene regulation for operons, genes in which are not transcriptionally independent to each other (Conway et al., 2014; Del Duca et al., 2023).

Compared to gene-based analyses, taking TUs as the basic analytic unit, often shows merits in bacterial comparative transcriptome studies. For example, transcriptomic comparison of *Salmonella* in planktonic and aggregate states revealed significant difference for more than 1,000 genes (MacKenzie et al., 2015). The number may have overstated actual biological variation, as some genes are co-regulated as parts of very long operons (Wang et al., 2015). Consequently, functional enrichment analysis solely on the differential genes could lead to misleading interpretations. In contrast, TU-based analysis not only reduces the number of comparisons, thereby increasing the statistical power (Xiao et al., 2006; Creecy and Conway, 2015; Won et al., 2024), but may also help identify differential functional pathways more precisely (Zaidi et al., 2021). By shifting the unit from genes to TUs, we can more directly and comprehensively understand how genes are collectively regulated at a systemic level (Ermolaeva, 2001). More importantly, with TUs as the basic components, it is more straightforward and simplified to observe the evolution of gene organization and regulation, capture a global picture about gene co-expression and co-regulation, and understand the hierarchical mechanisms for the organization, coordination and regulation of genes responsible for specific phenotypes. Dynamic regulatory characteristics of operons further demonstrate the value to take a TU as a basic unit for analysis. Recently, some studies disclosed dynamic organization of operons, that is, alternative transcription start and termination sites, and gene composition for the same putative operon under various conditions (Mao et al., 2015; Alfonso-Gonzalez and Hilgers, 2024). This complexity indicates that operons are not merely physical clusters of genes but also fundamental units of functional regulation. By analyzing the dynamic regulatory patterns of operons, we can gain deeper understanding of how bacteria adapt to environmental changes through coordinated gene expression.

Despite the potential advantages of TU-based analyses, systems-level studies on TUs remain relatively scarce, partially due to the limited experimental data on TU annotation and the methodology in TU identification. However, to date, as a large amount of gene expression data accumulate and we have in-depth understanding of genomic information in model bacteria, it is possible to explore the organization and interactions of bacterial TUs in the systems level (Salgado et al., 2000; Cao et al., 2019). To test the possibility, in this study, we took *S*. typhimurium as a model organism, integrated their genome and transcriptome data, and made a systematic survey on the TUs. It should be noted that, in this study, TUs could be more precisely defined as transcriptional regulation units, which contain both operons and singleton genes that are transcribed and regulated individually and independently. In fact, many operon-detecting tools also report the TUs, including both operons and independently transcribed singleton genes (Taboada et al., 2018; Krishnakumar and Ruffing, 2022; Tomar et al., 2023; Karaji and Peña-Castillo, 2025).

*Salmonella* is an important zoonotic pathogen. The genus consists of two species, *S. bongori* and *S. enterica*. *S. enterica* has evolved into more than 2,000 serovars (Winfield and Groisman, 2004), among which *S*. typhimurium is one of the most well-known representative broad-host-range pathogen (Galán, 2021). It has been widely used as a model organism for *Salmonella* pathogenicity study, and a large amount of omics and experimental data have been accumulated for *S*. typhimurium. However, we still have very limited knowledge about the systematic organization and regulation of genes for the organism. For example, the SPI-1 type III secretion system (T3SS) and the SPI-4 T1SS, together with the substrate proteins, were found to coordinate to facilitate the adhesion and invasion of *Salmonella* into host cells (Gerlach et al., 2007). However, the genes encoding these proteins are located in different operons scattered in the genome. It remains an enigma how these operons are co-regulated. Similarly, it is also unclear how the scattered operons encoding SPI-1 T3SS and the substrates are co-regulated with those encoding flagellar proteins, curli, and others (MacKenzie et al., 2015).

In this study, with *S*. typhimurium as the model organism, we proposed a transcriptome data based TU-detecting strategy, systematically identified the TUs, and investigated their evolution and regulation. The methodologies introduced here are also applicable to other bacteria, offering novel insights and perspectives for bacterial systems biology studies.

## Results

### Systematical identification of TUs in *S*. typhimurium

**Figure 1:**
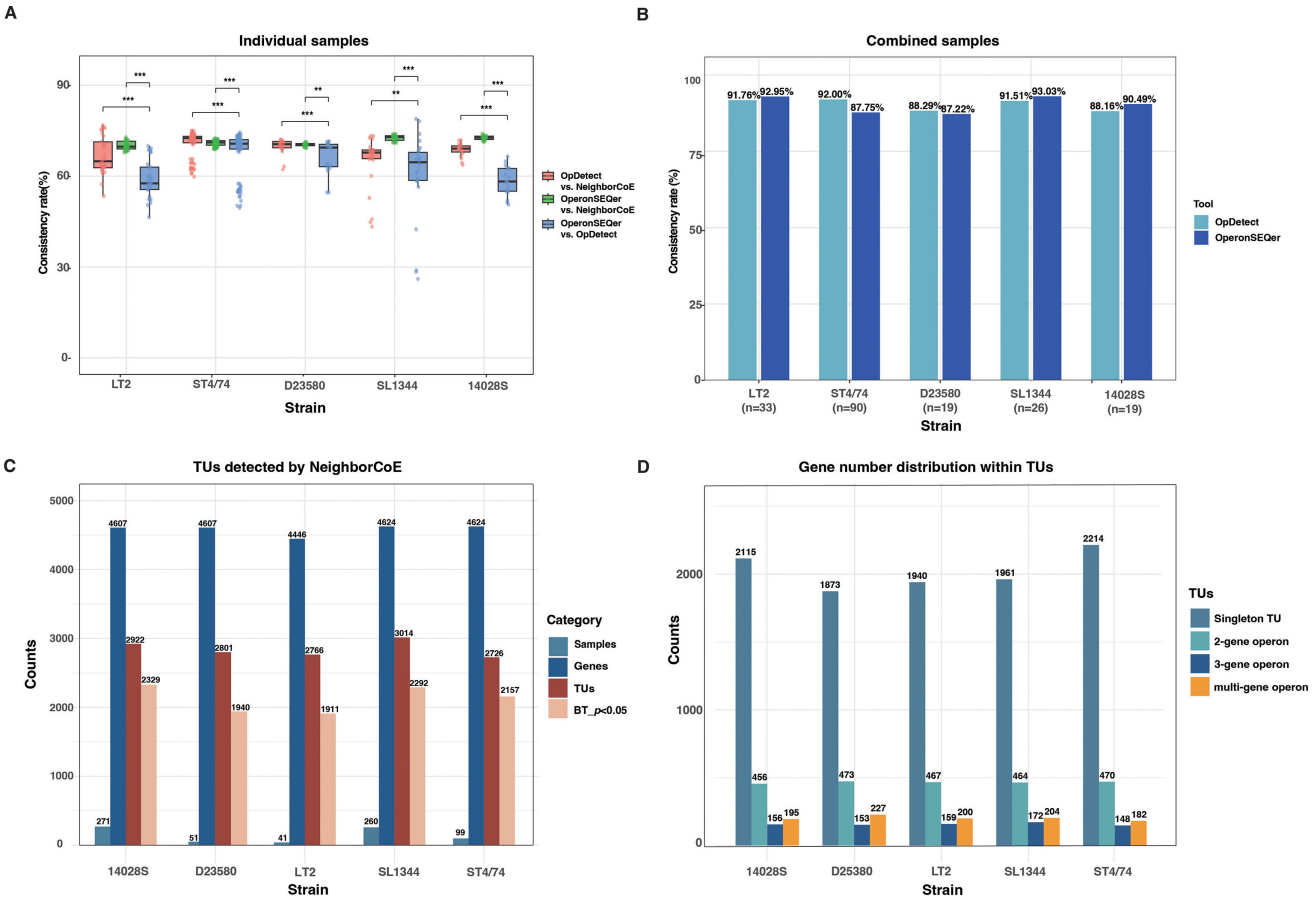
Performance of NeighborCoE in identification of the *S*. typhimurium operons and singleton TUs. **(A)** Consistency of TUs detected by different methods within individual samples. The consistency rates were compared between the method pairs with Wilcoxon rank-sum tests, and the significance was indicated. *p < 0.05; **p < 0.01; ***p < 0.001. **(B)** Consistency of TUs detected by different methods within multiple samples. For each strain, a random number (*n*) of RNA-seq samples were selected and indicated. **(C)** Summary of the TUs detected by NeighborCoE. The high-quality RNA-Seq datasets were downloaded from the NCBI SRA database, for five *S*. typhimurium strains (14028S, SL1344, LT2, ST4/74, and D23580). The total number of identified TUs, and the count of high-confidence TUs (bootstrapping test *p* < 0.05, abbreviated as BT_*p* < 0.05) were also shown. **(D)** The number and proportion of Singleton TUs, two-gene operons, three-gene operons, and multi-gene operons (with more than three genes) across different strains.

In this study, we proposed a semi-empirical strategy, NeighborCoE, to identify TUs based on both the locus neighborhood and expression correlation features (Section Materials and methods). We collected a total of 271 high-quality RNA-seq datasets for *S*. typhimurium strain 14028S, 260 for SL1344, 41 for LT2, 99 for ST4/74, and 51 for D23580 under diverse experimental conditions (Supplementary Table S1). With these strains and the gene expression data, firstly, we compared NeighborCoE with other state-of-the-art TU-identification tools such as OpDetect (Karaji and Peña-Castillo, 2025) and OperonSEQer (Krishnakumar and Ruffing, 2022). Different from NeighborCoE, both OpDetect and OperonSEQer mainly detect TUs within individual rather than combined samples. Within the randomly selected samples, the TUs detected by OpDetect and OperonSEQer showed median consistency rates of 64.9%−72.6% and 69.7%−72.9% for the five strains with the operons identified by NeighborCoE, respectively, while the consistency rates between the two tools were significantly lower, only reaching 57.6%−70.7% (Figure 1A; for all comparisons, Wilcoxon rank-sum testing *p* < 0.005). When the TUs identified from individual samples were combined, the consistency rates of operons detected by NeighborCoE and OpDetect or OperonSEQer were significantly increased, reaching or being larger than 90% (Figure 1B). Therefore, the TUs identified with the strategy of gene neighborhood and expression correlation in the *S*. typhimurium strains are reliable.

With NeighborCoE and the RNA-seq datasets, we identified 2,922, 3,014, 2,766, 2,726, and 2,801 TUs from the genome-coding genes of the *S*. typhimurium strains 14028S, SL1344, LT2, ST4/74 and D23580, respectively (Figure 1C; Supplementary Table S2). The TUs were also identified robustly, 69.1%−79.7% among which were supported by bootstrapping values greater than 95% (Figure 1C). The slightly reduced stability observed in LT2 and D23580 could possibly be due to the limited size of transcriptomic datasets (Figure 1C).

The majority of the TUs are composed by single genes, ranging from 1,873 to 2,214 (68.3%−73.5%) across the strains (Figure 1D; Supplementary Table S2). In contrast, two-gene, three-gene and multi-gene operons are relatively fewer, accounting for 456–473 (15.6%−17.4%), 148–172 (4.9%−6.1%), and 182–227 (6.0%−8.3%) of the total TUs, respectively (Figure 1D; Supplementary Table S2).

### Comparison of the TUs among *S*. typhimurium strains

**Figure 2:**
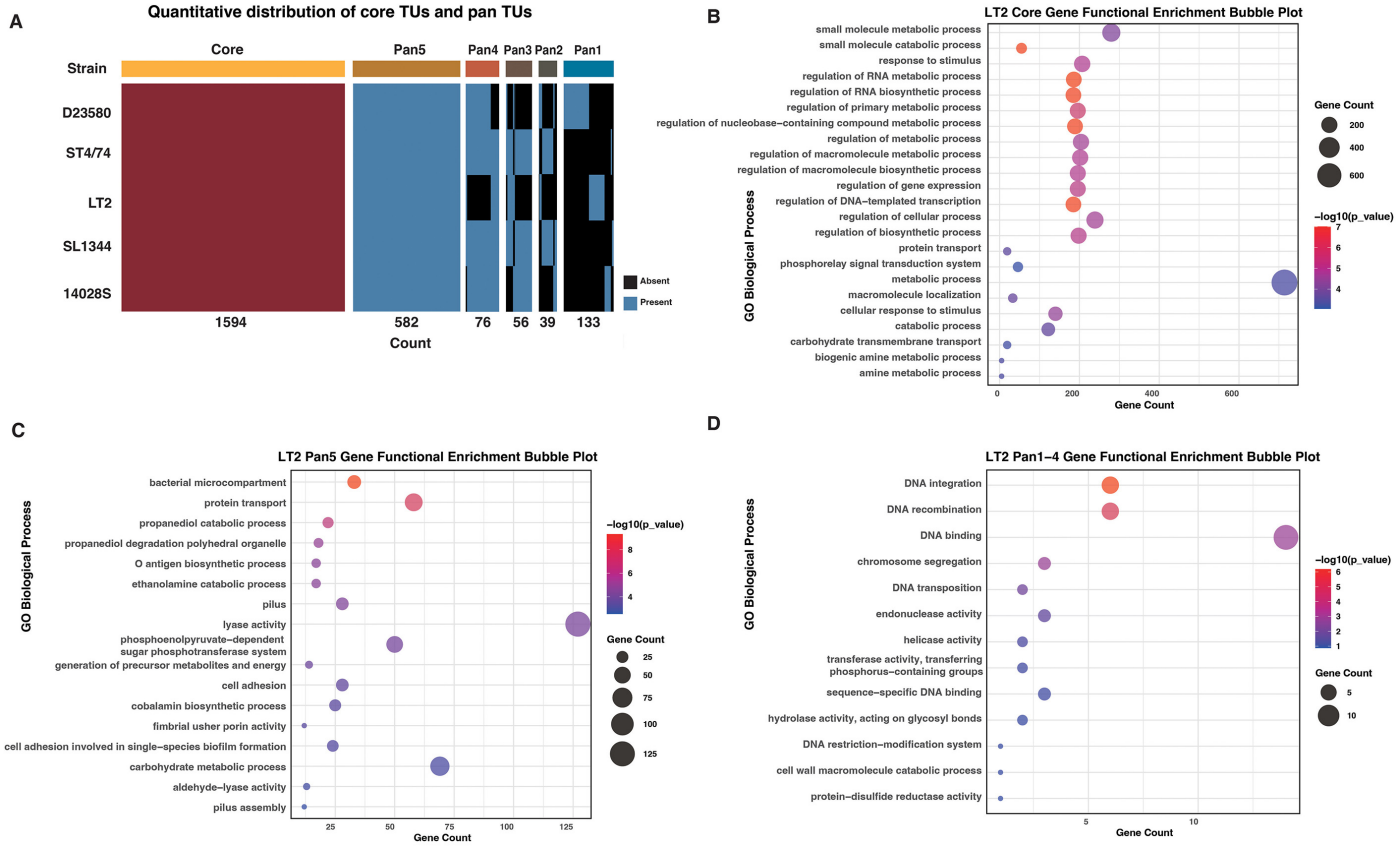
Comparative and functional enrichment analysis of TUs in *S*. typhimurium. **(A)** Quantitative distribution of core TUs and pan TUs (Pan1–Pan5) across five *S*. typhimurium strains. **(B–D)** Gene Ontology functional enrichment analysis of genes constituting Core **(B)**, Pan5 **(C)**, and Pan1-4 TUs in LT2 **(D)**.

The core TUs were identified for the five *S*. typhimurium strains by comparative analyses. In total, we identified 1,594 core TUs, which are strictly conserved across all the strains with identical gene content and synteny (Supplementary Table S3). The remaining non-core operons were grouped into 886 pan-TU families (or networks), with a network-based strategy (Section Materials and methods; Supplementary Table S3). Among the pan-TU families, the majority (582, designated as Pan5) are present in all five strains but displayed variations in gene composition or organization (Figure 2A; Supplementary Table S3). A smaller proportion of pan-TU families were shared by subsets of strains, including 76 in four (Pan4), 56 in three (Pan3), and 39 in two strains (Pan2; Figure 2A; Supplementary Table S3). Additionally, 133 TUs were identified as strain-specific (Pan1; Figure 2A; Supplementary Table S3). Taken together, the results indicate that, while most *S*. typhimurium TUs are conserved among strains, they retain plasticity that may facilitate strain-specific environmental adaptation.

Genes within the core TUs are enriched in functioning in bacterial essential cellular processes such as metabolism and transcription (Figure 2B). Besides the basic function, such as protein transport, the Pan5 TUs are also enriched with genes with other function, such as the synthesis and regulation of bacterial microcompartments, O antigen biosynthesis, pilus assembly, cell adhesion, and fimbrial assembly (Figure 2C). In striking contrast, TUs restricted to subsets of strains, that is, Pan1–Pan4 ones, are mainly associated with DNA recombination, integration, and transposition (Figure 2D). Collectively, these results suggest that the core TUs are highly conserved and indispensable for fundamental bacterial physiology, Pan5 TUs exhibit greater regulatory flexibility to accommodate environmental diversity, while strain-specific TUs may be likely dispensable for essential processes and instead contribute to specialized biological traits or represent recently acquired genes.

### Evolution of the operons in *S*. typhimurium

**Figure 3:**
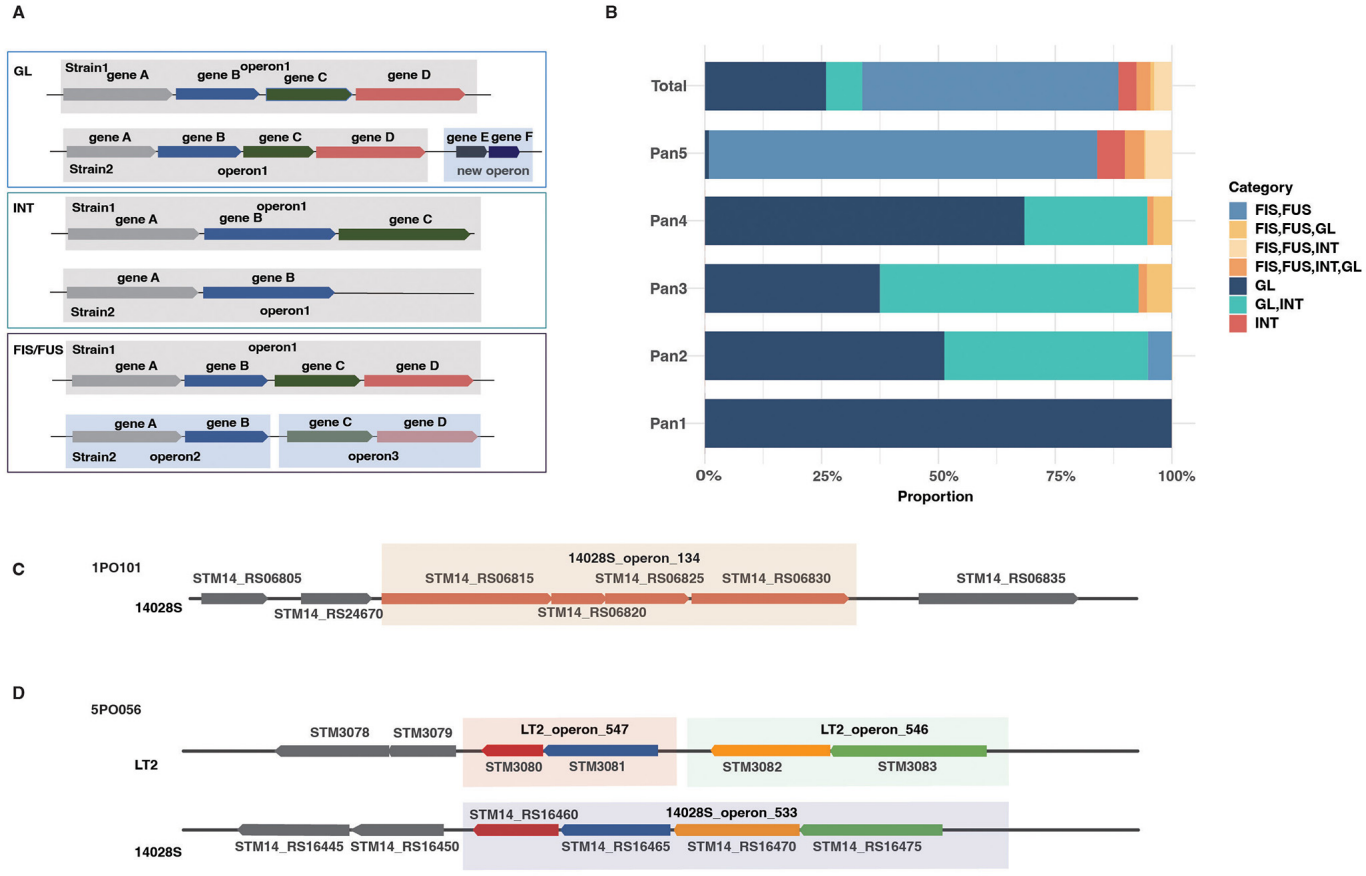
Evolutionary patterns of *S*. typhimurium operons. **(A)** Schematic representation of three major types of operon structural variations, including whole-operon gain or loss (GL), internal gene insertion or deletion (INT), and Operon fission or fusion (FIS/FUS). **(B)** Statistical distribution of operon variation types in *S*. typhimurium. **(C)** An example of GL-type variation: 14028S_operon_134 (containing four genes of unknown function) specifically acquired by strain 14028S via horizontal gene transfer. **(D)** An example of FIS/FUS-type variation (alternative operon organization, AO): 14028S_operon_533 in strain 14028S undergoing fission and reorganization into two adjacent operons, LT2_operon_547 and LT2_operon_546, in strain LT2.

Comparison of variable members within the pan-TU families revealed three primary evolutionary patterns of operons in *S*. typhimurium, including (1) Whole-operon Gain or Loss (GL), (2) Internal gene insertion or deletion (INT), and (3) Operon Fission or Fusion (FIS/FUS; Figure 3A). Many operon families exhibit mixed patterns, characterized by the simultaneous presence of multiple distinct variation modes (Figure 3B). Generally, we identified 333 GL (37.4%), 162 INT (18.2%), and 556 FIS/FUS events (62.5%) from the *S*. typhimurium pan-TU families (Figure 3B).

A single gene rearrangement event can give rise to multiple forms of operon variation. For instance, a horizontally acquired gene may itself form a GL-type operon, while its insertion site may simultaneously cause an INT-type variation or lead to the fission/fusion of an affected operon (Figure 3A, INT). We also distinguished a subset of FIS events, causing Alternative Operon Organization (AO), which involves reorganization of shared genes without the insertion or loss (Figure 3A, FIS/FUS). We gave two typical examples to show the GL-type variation and the AO-causing FIS/FUS-type variation, respectively. 1PO101 is an example for the former while 5PO056 illustrates the latter pattern (Figure 3C). Within the 1PO101 family, there is only a four-gene operon, 14028S_operon_134, which is totally absent from the other *S*. typhimurium strains for all the composition genes and thus represents the GL-type variation among strains (Figure 3C). For the 5PO056 family, the single operon 14028S_operon_533 in strain 14028S is split into two adjacent operons (LT2_operon_547 and operon_546) in LT2, illustrating a typical case of FIS/FUS-type variation among strains (Figure 3D).

It should be noted that the vast majority of inter-strain differences in the pan TU, mainly in Pan5, with the largest number of pan-TU families, are not driven primarily by the large-scale horizontal transfer of entire operon clusters, but are instead mediated by the architectural variation of otherwise conserved genes, predominantly through FIS/FUS events (Figure 3B). Therefore, we propose that AO, rather than simple gene gain or loss, serves as a primary mechanism for adaptive evolution in *S*. typhimurium, in response to specific environmental challenges and metabolic demands.

### TU co-expression network of *S*. typhimurium

**Figure 4:**
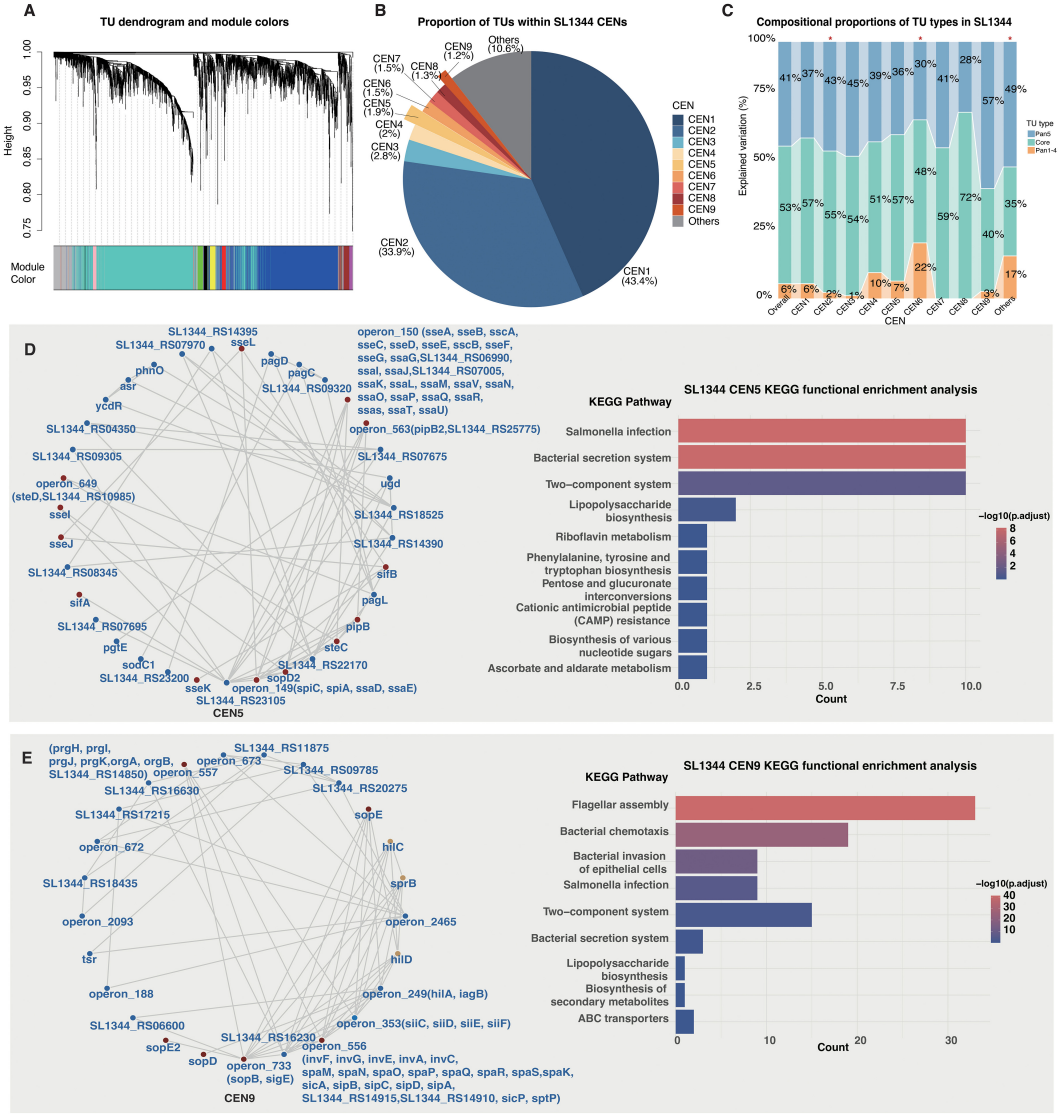
SL1344 CENs. **(A)** Hierarchical clustering dendrogram of TU co-expression modules identified by WGCNA. **(B)** Proportion of TUs within nine co-expression modules (CEN1–9) and the unclassified modules (others). **(C)** Compositional proportions of TU types (Core, Pan5, and Pan1–4) in each CEN module. Chi-square tests were performed between each module and the overall network for the distribution of TU groups, and each module with significant difference was indicated with an asterisk over the bar. **(D, E)** KEGG functional enrichment analysis of genes within the TUs of CEN5 **(D)** and CEN9 **(E)**, respectively. The singleton TUs were represented by their gene names while each operon was represented by the operon identification. For operons with T3SS-related genes, the gene names were also indicated.

Based on the available RNA-seq data, we re-quantified the expression of SL1344 TUs and calculated the pairwise correlation coefficients between operon pairs. Generally, subnetworks are hardly separated with a cutoff-based strategy, since the operons show large correlation coefficients between each other and the overall network is compactly connected. Alternatively, we employed Weighted Gene Co-expression Network Analysis (WGCNA) to build and decompose the overall TU Co-Expression Network (CEN) in to nine separated modules, namely CEN1 through 9 (Figure 4A; Supplementary Table S4). The CENs comprise 1,307 (43.4%), 1,022 (33.9%), 83 (2.8%), 59 (2.0%), 58 (1.9%), 46 (1.5%), 46 (1.5%), 40 (1.3%), and 35 (1.2%) TUs, respectively (Figure 4B; Supplementary Table S4). Only 318 (10.6%) TUs could not be assigned to any specific CEN and were recognized as “Others” (Figure 4B).

To characterize the genomic composition of these networks, we re-categorized the TUs within each CEN into the Core- and Pan-TU groups (Core, Pan5, and Pan1–4). By comparing the distribution of TU groups within each CEN module against the global TU pool, we found that CEN2 (Chi-Squared Test, χ^2^ = 18.91, *p* = 7.84e−05), CEN6 (χ^2^ = 20.10, *p* = 4.31e−05) and the “Others” modules (χ^2^ = 72.97, *p* = 1.43e−16) exhibit significant differences in their TU-type composition (Figure 4C). We further consolidated the TU types into two categories, Pan1–4 and non-Pan1–4 (Core and Pan5), to refine the comparison. Significance was observed consistently for CEN2 (Fisher's exact test, *p* = 4.27e−06), CEN6 (*p* = 3.37e−04) and ‘Others' (*p* = 2.47e−10), suggesting that these differences are primarily driven by the proportion of Pan1–4 TUs. Specifically, CEN2 is significantly depleted of Pan1–4 TUs, whereas CEN6 and “Others” are significantly enriched for them (Figure 4C). It implies that CEN2 likely represents a basal functional network, while CEN6 and “Others” are predominantly composed of accessory TUs. CEN1 contains the largest number of TUs, and the TU-type distribution does not deviate from the overall pool (Figure 4C), suggesting it serves as a housekeeping network closely tied to fundamental bacterial life processes. For the other modules, even some show apparent difference for the proportions of different TU types, for example, CEN7 and CEN8 with low proportions of Pan1–4 TUs (Figure 4C), and yet the differences are not significant, potentially due to the much smaller module size (Figure 4B).

Functional enrichment analysis of the genes within the TUs in each CEN demonstrates that, the genes in CEN1, 2, 3, 4, 7, and 8 are all enriched for basic biological function such as metabolism, protein transport, and signal transduction (Supplementary Figure S1). In contrast, CEN6 is enriched for genes related to LPS synthesis and *Salmonella* infection, consistent with its higher proportion of Pan1–4 TUs (Supplementary Figure S2). The “Others” group is associated with microbial metabolism in diverse environments, potentially implying a high prevalence of horizontally transferred genes for better strain-specific adaptation (Supplementary Figure S2). Although the TU-type distribution of CEN5 or CEN9 does not differ significantly from the global distribution, gene annotation reveals distinct virulence-related roles. CEN5 contains a high density of genes encoding *Salmonella* Pathogenicity Island 2 (SPI-2) T3SS components and their associated effectors (Figure 4D), whereas CEN9 is enriched for gene encoding the SPI-1 T3SS and effectors (Figure 4E). Genes in CEN9 are also enriched for flagellar assembly, bacterial chemotaxis, and bacterial invasion of epithelial cells (Figure 4E). Together with the differential function of SPI-1 and SPI-2 T3SSs, the results suggest that CEN9 could be a network predominantly participating in *Salmonella* invasion, while CEN5 function specifically in bacterial intracellular survival and systemic infection.

### TU transcriptional regulation network of *S*. typhimurium

Furthermore, we tried to characterize the transcriptional regulatory landscapes of the identified CENs in *S*. typhimurium. By curating ChIP-seq datasets for various transcriptional regulators, we annotated 23 regulators, yielding 2,828 high-confident regulator-TU regulon pairs (Supplementary Table S5). By mapping the TUs within each regulon to their respective CENs, we reconstructed the specific regulatory networks for each co-expression module.

**Figure 5:**
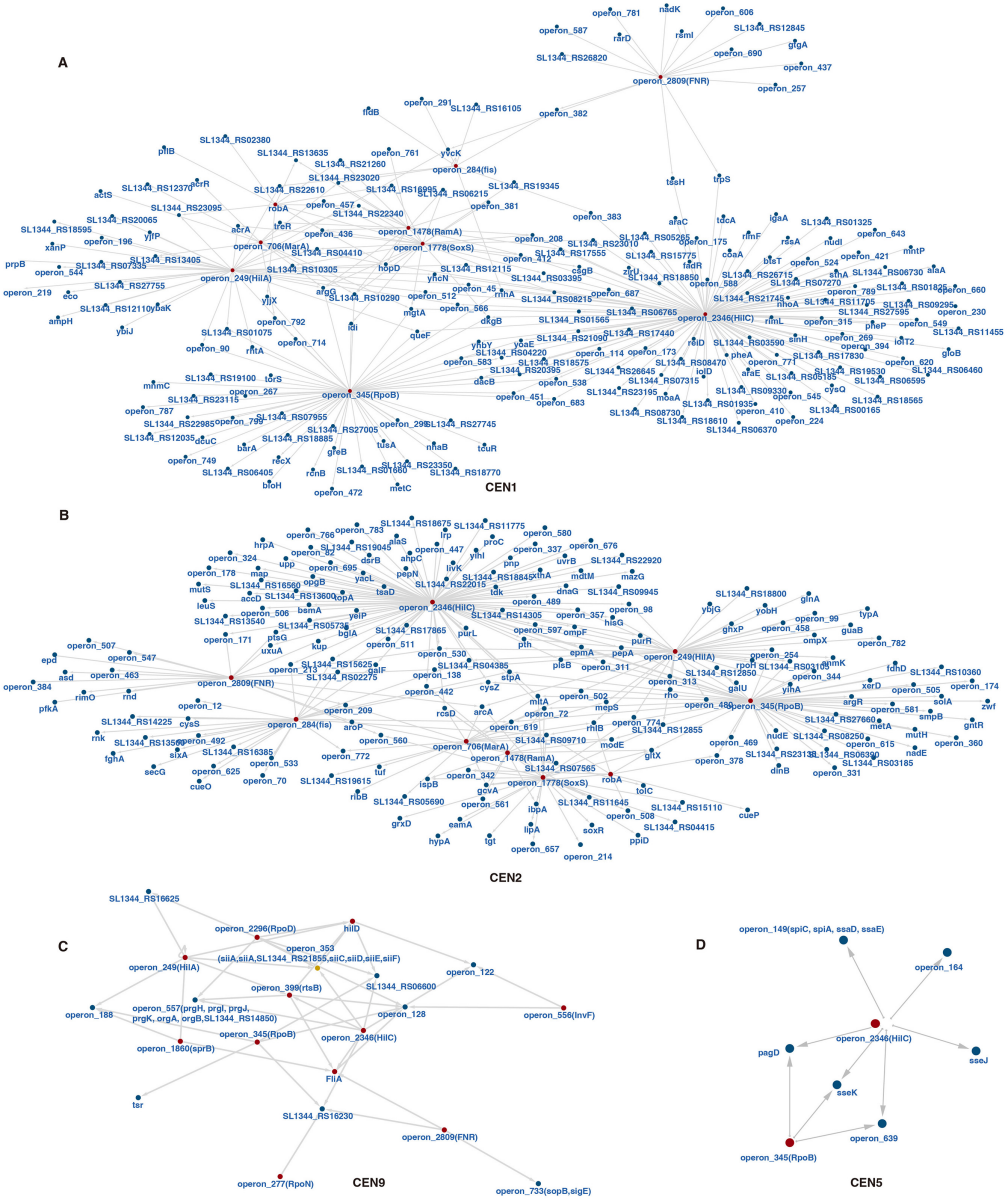
Inferred transcriptional regulatory circuits for representative CEN modules in SL1344. **(A–D)** The regulatory network for CEN1 and CEN2, two modules involving bacterial basic metabolic pathways **(A, B)**, virulence-related CEN9 **(C)** and CEN5 that could participate in systemic infection **(D)**. The regulators and SPI-1/2 T3SS related genes were indicated.

Although the annotated regulators only comprise a limited subset, which are mainly associated with pathogenicity and adaptation, large regulatory networks were identified within CEN1 and CEN2 (Figure 5A). The regulators also form distinct regulatory circuits in other CENs, modulating multiple downstream TUs (Supplementary Figure S3). Some of the key regulators, for example, HilA and HilC, do not merely regulate virulence-related TUs but are also deeply integrated into the bacterial systemic regulation. In CEN9, we identified a dense regulatory network connected by multiple hub regulators (Figure 5B). Regulons encode SPI-1 T3SS components and their effectors, SPI-4 Type 1 Secretion System (SiiCDEF), and other critical virulence factors (Figure 5B). The key hub regulators include HilA, HilC, HilD, RpoB, RpoD, and RtsB (Figure 5B). Interestingly, HilA and RpoB are also present in the circuit that regulate operons within CEN5 (Figure 5C). The majority of the regulons in CEN5 encode SPI-2 T3SS components and the effectors (Figure 5C).

### Function of CEN9 and CEN5 in *S*. typhimurium

TU co-expression analysis detected two modules closely related with the two sets of *Salmonella* T3SSs, that is, CEN9 associated with the SPI-1 T3SS activity, and CEN5 associated the SPI-2 T3SS activity. It is interesting to explore whether there are more new Type III Secreted Effectors (T3SEs). We therefore make a genome-wide *de novo* T3SE prediction in SL1344 with two state-of-the-art (SOTA) models, T3SEpp and CLEF, followed by analysis on the intersections between T3SEpp/CLEF results and the genes within CEN9/5. T3SEpp and CLEF predicted 56 and 187 T3SEs from the SL1344 genome, respectively.

**Figure 6:**
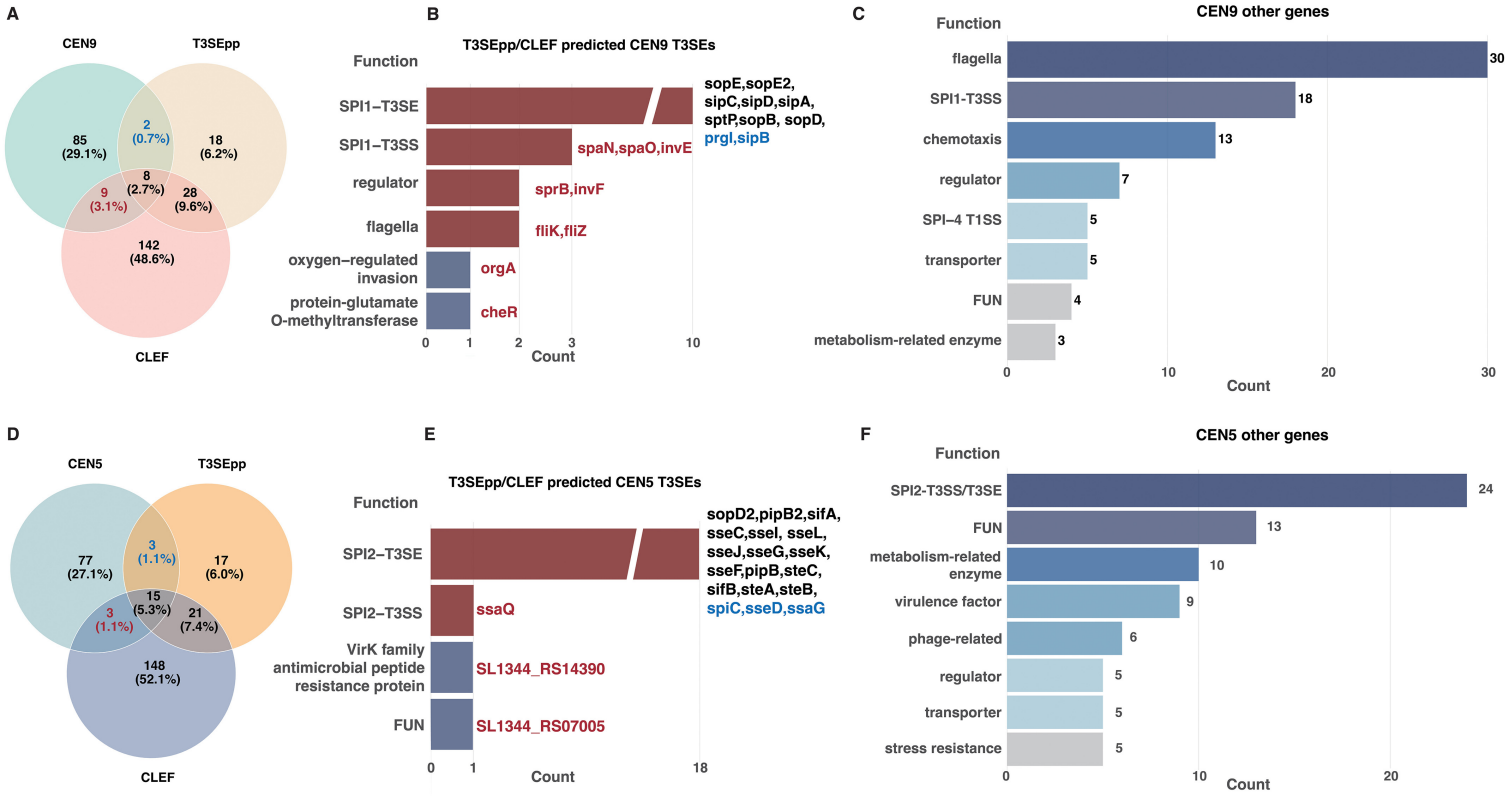
Functional exploration of the SL1344 CEN9 and CEN5 modules. **(A, D)** Venn diagrams showing the overlap between T3SE genes predicted by sequence-based models and the genes in CEN9 **(A)** or CEN5 **(D)**, respectively. **(B, E)** Annotations of the CEN9 **(B)** or CEN5 **(E)** genes predicted to be T3SEs by the models, respectively. The genes specifically predicted by T3SEpp and CLEF were shown in blue and red, respectively. **(C, F)** Functional annotation of the remaining genes in CEN9 **(C)** and CEN5 **(F)** modules not predicted to be T3SEs by the models.

The total 104 genes in CEN9 show an overlap of 10 with T3SEpp predictions and 17 with CLEF, with eight genes shared by all three sets (Figure 6A; Supplementary Table S6). All the eight genes in the three-way intersection encode known SPI-1 T3SEs (Supplementary Table S6). Two additional CEN9 genes predicted solely by T3SEpp (*sipB* and *prgI*) also encode known SPI-1 T3SEs (Figure 6B; Supplementary Table S6). Among the nine genes predicted exclusively by CLEF in CEN9, three encode putative T3SS structural components (*spaN, spaO*, and *invE*), two encode transcriptional regulators (*sprB* and *invF*), two encode flagella-related genes (*fliZ* and *fliK*), and two encode virulence-related factors (*orgA* and *cheR*) (Figure 6B). Consequently, no novel T3SEs were identified in CEN9 using these tools. Among the remaining 85 genes in CEN9, 81 have well-defined functional annotations related to *Salmonella* virulence and invasion, but do not encode T3SEs (Supplementary Table S7). Only four genes remain uncharacterized, yet neither T3SEpp nor CLEF identified T3SE-like features in the sequences, making them unlikely candidates for novel T3SEs (Figure 6C).

CEN5 comprises 98 genes in total, with 18 genes overlapping with T3SEpp, 18 with CLEF, and 15 with the predictions of both models (Figure 6D; Supplementary Table S6). Similar to the findings in CEN9, all the 15 genes in the three-way intersection encode known SPI-2 T3SEs (Supplementary Table S6). The three CEN5 genes predicted solely by T3SEpp (*spiC, sseD*, and *ssaG*) are also known SPI-2 T3SEs (Figure 6D; Supplementary Table S6). Among the three genes predicted exclusively by CLEF, *ssaQ* encodes a structural component of the SPI-2 T3SS, and *SL1344_RS14390* encodes a VirK-family antimicrobial peptide resistance protein (Figure 6E). Only one gene, *SL1344_RS07005*, remains functionally uncharacterized and the possibility that it encodes a T3SE cannot be excluded (Figure 6E). Among the remaining 77 genes in CEN5, 58 have clear annotations related to systemic infection and stress adaptation (Supplementary Table S7). Aside from three known T3SEs that both T3SEpp and CLEF failed to predict (*ssaI, sseB*, and *steD*), there are six genes of prophage origin and 13 remain functionally uncharacterized (Figure 6F, Supplementary Table S7).

In summary, CEN9 could potentially be a module closely associated with *S*. typhimurium invasion and virulence. The majority of genes in this module include those encode the SPI-1 T3SS components, effectors and regulators, which are most well-known for functioning in *Salmonella* invasion. Other gene products, such as the SPI-4 T1SS and the substrate, flagella components and various virulence factors, also function synergistically with the SPI-1 T3SS to participate in bacterial invasion and virulence, but are unlikely to encode novel SPI-1 T3SEs. CEN5, on the other hand, is related to systemic infection and intracellular survival, including the SPI-2 T3SS and its effectors, as well as two-component systems and other stress factors that may function together with the SPI-2 T3SS to maintain bacterial intracellular survival, though the probability of genes within the module encoding new T3SEs is also low.

## Discussion

TU-based analysis in bacterial systems biology studies at the transcription level has many merits compared to gene-centric analysis such as the higher accuracy, reduced information redundancy and improved statistical power (Wells et al., 2016; Métris et al., 2017; Bundalovic-Torma et al., 2020; Olbei et al., 2022), though the majority of bacterial transcriptome studies still rely on genes as the primary unit (Métris et al., 2017; Olbei et al., 2022). Here, we took *S*. typhimurium as a model organism to make a TU-based systems biology study. Specifically, we integrated *S*. typhimurium genomic information with large-scale RNA-seq data to perform genome-wide identification of TUs, including both operons and independently transcribed single genes, followed by comparative TU analysis, and characterization of the evolutionary patterns of TUs, the co-expression networks and regulatory networks.

We observed that the core TUs of *S*. typhimurium are conserved and play critical roles in fundamental metabolic processes (Figure 2B). The gene composition and genomic organization of pan-operons exhibit significant variability. Across different strains, the constituent genes within the same pan-operon family may vary, but on the other hand, an identical set of genes may assemble into distinct operon structures. Recently, it has been recognized that bacterial operons exhibit variable organization, which is often related to bacterial pathogenicity and environmental adaptation (Mao et al., 2015; Warrier et al., 2018; Yan et al., 2018). We also noticed that pan-TU families conserved across a larger number of strains are more frequently involved in fundamental biological processes, whereas those exhibiting higher variability among strains are largely associated with horizontal gene transfer and recombination, which could be more closely related with strain-specific phenotypes (Figures 2C,D).

The *S*. typhimurium global TU CEN appears very compact and difficult to decompose into discrete sub-networks due to the universally strong correlations between the TUs. It suggests that gene expression in *Salmonella* is likely subject to strict systemic regulation, necessitating that horizontally acquired genes coordinate with the global expression landscape. Alternatively, the global regulation and stability of gene expression may serve as a critical constraint on genomic recombination. Recently, utilizing ancestral genome reconstruction combined with multi-omics analysis, we confirmed that the *Salmonella* genome is highly conserved, a trait closely intertwined with global gene expression and chromosome conformation (Hu et al., 2024). We further employed WGCNA to decompose the CEN. The two primary modules (CEN1 and CEN2) encompass the absolute majority of TUs (2,329/3,014, 77%). Not surprisingly, genes within these modules are primarily involved in fundamental functions such as metabolism (Supplementary Figures S1A, B). The remaining TUs cluster into seven smaller modules (CEN3–9), among which CEN9 and CEN5 are of particular interest. CEN9 harbors genes encoding the SPI-1 T3SS components and effectors, as well as the SPI-4 T1SS, flagella, and other virulence factors (Figure 6E). Both SPI-1 T3SS and SPI-4 T1SS are well-known to be closely associated with *Salmonella* invasion (Gerlach et al., 2007). Given that T3SSs origin from flagellar systems (Macnab, 2004; Diepold and Armitage, 2015), and that *Salmonella* flagellin genes are frequently co-expressed with SPI-1 T3SS genes (MacKenzie et al., 2015), the enrichment of these elements, along with other virulence factors, suggests that CEN9 likely constitutes a distinct functional entity driving host invasion. In contrast to CEN9, CEN5 contains genes encoding SPI-2 T3SS components and substrates, alongside two-component systems and other stress responsors essential for bacterial *in vivo* survival. Thus, CEN5 is likely involved in systemic infection and intracellular survival (Figure 5D). We attempted to predict SPI-1/2 T3SEs within CEN9 and CEN5 but failed to identify candidates. This may indicate that the repertoire of *Salmonella* T3SEs has been largely characterized and few new T3SEs remain to be discovered, although we cannot exclude the possibility that there could be new T3SEs present in other modules.

We also systematically integrated ChIP-seq data of *Salmonella* transcriptional regulators to identify their respective operon regulons. Beyond the large networks identified in CEN1 and CEN2, CEN9 exhibits a notably complex regulatory architecture (Figures 5A,B). HilA, HilC, HilD, RtsB, RpoD, InvF, and others form an intricate network that co-regulates the SPI-1 T3SS (and its effector genes), SPI-4 T1SS, flagella, and other virulence genes (Figure 5B). Previous studies have established that the SPI-4 T1SS and SPI-1 T3SS in *Salmonella* are highly co-expressed and function synergistically in bacterial adhesion and invasion (Gerlach et al., 2007; Yu et al., 2025). The regulatory network uncovered here provides a more systematic mechanistic explanation for this coordinated expression. The regulatory network for CEN5 remains less defined, likely due to the limited number of regulators with available ChIP-seq data (Figure 5C).

The current study also has some limitations. First, the accuracy of TU identification is heavily dependent on the sample size of gene expression data. Currently, abundant transcriptomic data are only available for a few *S*. typhimurium strains, whereas data for serovars more closely associated with human disease, for example, *S*. typhi, *S*. paratyphi A, and invasive Non-Typhoid Salmonellae (iNTS) strains, remain limited. The extent to which the TUs identified in *S*. typhimurium can be transferred and applied to other *Salmonella* serovars requires further exploration. Second, due to data scarcity, we were unable to accurately determine the TU-regulons for a broader array of transcriptional regulators. Consequently, the regulatory relationship within each CEN requires further clarification and refinement. In the future, integrating experimental data on transcriptional regulation of individual operons and regulatory information from homologous genes in other species could enhance the resolution of the regulatory networks. Finally, within a single strain, TU organization may also vary across different environmental conditions (Mao et al., 2015). Therefore, how to characterize this dynamic heterogeneity requires further exploration.

In conclusion, we proposed a scheme of TU-based systems biology analyses and leveraged genomic and transcriptomic data to systematically investigate the evolution, organization, and regulation of the *S*. typhimurium TUs. The analytic framework and methods are also applicable to other bacterial strains. The results accumulated in this study provide valuable resources and insights for advancing research in *Salmonella* systems biology, pathogenicity, and genome evolution.

## Materials and methods

### Datasets

Five representative strains of *S*. typhimurium (LT2, 14028S, SL1344, ST4/74, and D23580) were used. The complete sequences and annotation for their chromosomes and plasmids were obtained from the NCBI Genome database (https://www.ncbi.nlm.nih.gov/home/genomes/; Supplementary Table S1). A combined search strategy was applied in the NCBI GEO database (https://www.ncbi.nlm.nih.gov/geo/) using the keywords “Salmonella” AND “RNA sequencing”/“RNA-seq,” restricting the species to the aforementioned strains. Raw RNA-seq data for these strains under various experimental conditions and host environments were screened and downloaded from the SRA database (https://www.ncbi.nlm.nih.gov/sra; Supplementary Table S1). ChIP-seq experiments for *S*. typhimurium transcriptional regulators were searched from PubMed, while the raw sequencing data were retrieved from the SRA database (Supplementary Table S1).

For raw RNA-seq data, quality control was performed with low-quality bases and adapters removed using Trim Galore v0.6.10 (https://doi.org/10.5281/zenodo.7598955) with the parameters “-q 20 –phred33 –length 20 –gzip”; the “–paired” option was additionally used for paired-end libraries. Reads shorter than 20 bp after trimming were discarded. The high-quality clean reads were aligned to the corresponding reference genome using Bowtie2 v2.2.5 (Langmead and Salzberg, 2012) with the parameters “–phred33 –local –very-sensitive-local,” using the “-1” and “-2” options for paired-end reads and “-U” for single-end reads. The alignments were then sorted by read name and written in SAM format using SAMtools v1.18 (“sort -n -O sam”; Li et al., 2009). Gene-level read counts were generated using HTSeq-count v2.0.2 with the parameters “-f sam -r name -s reverse -a 10 -t CDS -i locus_tag -m intersection-strict” (Anders et al., 2015). Gene expression level was normalized using the RPKM (Reads Per Kilobase per Million mapped reads) method. For ChIP-seq data, quality control, adapter trimming, and read alignment were performed using the same software and parameters as described above for RNA-seq data. The low-quality samples with an alignment rate < 70% were excluded.

### Identification of operons and single-gene TUs

In this study, we proposed a new strategy, namely NeighborCoE, which integrated genomic adjacency and co-expression information to identify operons. For each *S*. typhimurium strain, co-directional adjacent gene pairs along the genome were extracted. Pearson correlation coefficient (PCC) was calculated between each pair of adjacent genes for their expression levels measured by log-transformed RPKM values. This operon identification strategy relies on two parameters, the intergenic distance (*d*) between adjacent genes and the PCC cutoff value (*c*) for expression correlation. Empirically, we applied a step-wise, optimized combination of *d* and *c* to identify operons based on the following criteria: (*d* ≤ 10 bp), or (*d* ≤ 50 bp and *c* > 0.5), or (*d* ≤ 100 bp and *c* > 0.6), or (*d* ≤ 200 bp and *c* > 0.7), or (*d* ≤ 500 bp and *c* > 0.8). NeighborCoE reports the full set of TUs, including both the operons and singleton TUs. To evaluate the robustness of TU identification, a bootstrapping test was performed. Specifically, subsets of the original RNA-seq samples were randomly resampled with replacement to create 100 iterations. For each of the 100 iterations, we independently repeated the entire TU identification process. TUs with a detection frequency of ≥95% were defined as high-confidence operons, which corresponds to a bootstrapping test *p* < 0.05, and the proportion of high-confident TUs was assessed subsequently.

Specifically, subsets of the original RNA-seq samples were randomly selected to repeat the operon identification process (100 iterations). TUs with a detection frequency of ≥95% were defined as high-confident TUs, and the proportion of high-confident TUs was assessed subsequently.

We also compared the NeighborCoE and other operon -identification methods including OpDetect (Karaji and Peña-Castillo, 2025) and OperonSEQer (Krishnakumar and Ruffing, 2022). The two methods predict both operons and singleton TUs from individual samples, and we randomly selected samples from the RNA-seq datasets of *S*. typhimurium strains. The tools were implemented to detect TUs from each sample with the default parameters. The detected TUs were compared between the two tools, and between them and NeighborCoE, followed by calculation of the average consistency rate of TUs per sample, respectively. The rates were compared between the tools with Wilcoxon rank-sum tests, with a preset significance level of *p* < 0.05. For either OpDetect or OperonSEQer, the union of TUs identified from individual samples were also analyzed for each strain, and then compared with the operons identified by NeighborCoE to calculate the consistency rates.

### Identification of core TUs and pan-TU networks

Panaroo (Tonkin-Hill et al., 2020) was employed to construct the pan-genome for the five *S*. typhimurium strains. The core gene families were also identified from the pan-genome results if they were presented in all the five strains. The core genes were subsequently mapped to their respective TUs. A TU was defined as a core TU only if all of its constituent genes were core genes, and both the gene composition and gene order were identical across all the strains. The non-core TUs in each strain were categorized into distinct cross-strain pan-TU network based on the pan-genome family identity of their constituent genes. Within each pan-TU network, TUs from individual strains were represented as nodes. Nodes were connected if they share at least one constituent gene from the same pan-genome family. Using LT2 as the representative strain, Gene Ontology (GO) functional enrichment analysis was performed on the constituent genes of each TU subset using the R package *clusterProfiler* v4.10.1 (Yu et al., 2012).

### Analysis of the CENs of *S*. typhimurium

Two strategies were used to calculate the expression correlation between TUs and to infer the CENs of *S*. typhimurium. The first approach calculated the expression correlation between the constituent genes of any given pair of TUs using the PCC of their log-transformed RPKM values. The median of all these gene-level PCC values was used to represent the overall PCC between the two TUs, followed by building of the overall CEN and decomposing it into subnetworks with a PCC cutoff-based method. For the second approach, read counts of the constituent genes were aggregated at the operon level to calculate operon-specific RPKM values. Weighted gene co-expression network analysis was performed using the WGCNA package (v1.73). The soft-thresholding power was selected using the pickSoftThreshold function based on the scale-free topology criterion. Modules were identified using blockwiseModules with the following parameters: TOMType = “unsigned,” maxBlockSize = ncol (WGCNA_matrix), minModuleSize = 30, deepSplit = 2, reassignThreshold = 0, mergeCutHeight = 0.25, and pamRespectsDendro = FALSE. Module labels were returned numerically, TOMs were saved, and reproducibility was controlled using a fixed random seed (Langfelder and Horvath, 2008). Cytoscape (ver. 3.1.1) was used to visualize the networks with default settings (Shannon et al., 2003). To further elucidate the biological functions of the co-expression modules, KEGG pathway enrichment analysis was conducted for each co-expression module using *clusterProfiler* v4.10.1 (Yu et al., 2012).

### Analysis of the TRNs of *S*. typhimurium

For the pre-processed ChIP-seq data, MACS v2.2.9.1 was employed alongside the corresponding strain genomes to identify the genomic binding regions of transcriptional regulators (Zhang et al., 2008). Significant binding peaks were called using default settings. A TU was defined as a regulon for a specific regulator if the binding peak was located within 800 bp upstream of the translation start codon for the first gene within the TU. For each identified regulator-regulon pair, both the regulator and the regulon were mapped to their orthologs and core/pan TU families across different *S*. typhimurium strains, respectively, so as to expand the repertoire of regulatory interactions. The resulting regulator-regulon pairs were then used to construct the TRNs, which were visualized with Cytoscape (ver. 3.1.1; Shannon et al., 2003).

### T3SE prediction with sequence-based models

The whole-genome encoded proteins of the *S*. typhimurium SL1344 were screened for T3SE candidates with two sequence-based models, T3SEpp (Hui et al., 2020) and CLEF (Peng et al., 2025), with respective default parameters. UniProt (https://www.uniprot.org/) was referred to, to annotate the function of the T3SE candidates or other proteins.

### Statistics

The distribution of TU categories between CEN modules was compared using Chi-squared tests. To further delineate the enrichment of specific TU categories within each CEN module, two-tailed Fisher's exact tests were performed. Statistical significance was preset as *P* < 0.05 for all the analysis. For KEGG and GO functional enrichment analysis, hypergeometric tests were employed. Multiple testing correction was performed with the Benjamini–Hochberg (BH) method (Benjamini and Hochberg, 1995), and the False Discovery Rate (FDR) was calculated. The significance was defined as FDR < 0.05 for all the enrichment analysis.

### Availability of codes

The bioinformatic analysis was implemented using the NeighborCoE pipeline developed in Python. Genomic features were extracted from GenBank (.gbff) and GFF (.gff) files utilizing the Biopython (v1.78) library (Cock et al., 2009). Custom scripts were employed to calculate intergenic distances, including boundary corrections for the circular genome topology. For transcriptomic data processing, raw read counts were normalized to RPKM using the *bioinfokit* package (v2.1.4) (http://doi.org/10.5281/zenodo.3698145). The scipy.stats module was subsequently used to calculate PCC for all adjacent gene pairs. The full source code for this study is publicly available at https://github.com/lokting/NeighborCoE and https://doi.org/10.5281/zenodo.19280036. Representative sample input and output files are provided in the “examples/” directory of the GitHub repository and the corresponding Zenodo archive, including example datasets for operon identification, core TU construction, and pan-TU analysis. Detailed step-by-step instructions for preparing input files, running each module, and locating the corresponding output files are provided in the README.md file. We also developed a web interface for annotating *S*. typhimurium TUs, which is available at https://tools.szu-bioinf.org/TU.

## Data Availability

The original contributions presented in the study are included in the article/Supplementary material, further inquiries can be directed to the corresponding author.
