# Supplementary figures and images for: A transcription unit based systems biology study on Salmonella typhimurium gene organization, evolution, co-expression, and regulation
Source: Front Microbiol. 2026 May 21;17:1816957. doi: 10.3389/fmicb.2026.1816957 (PMC13233679; doi:10.3389/fmicb.2026.1816957)

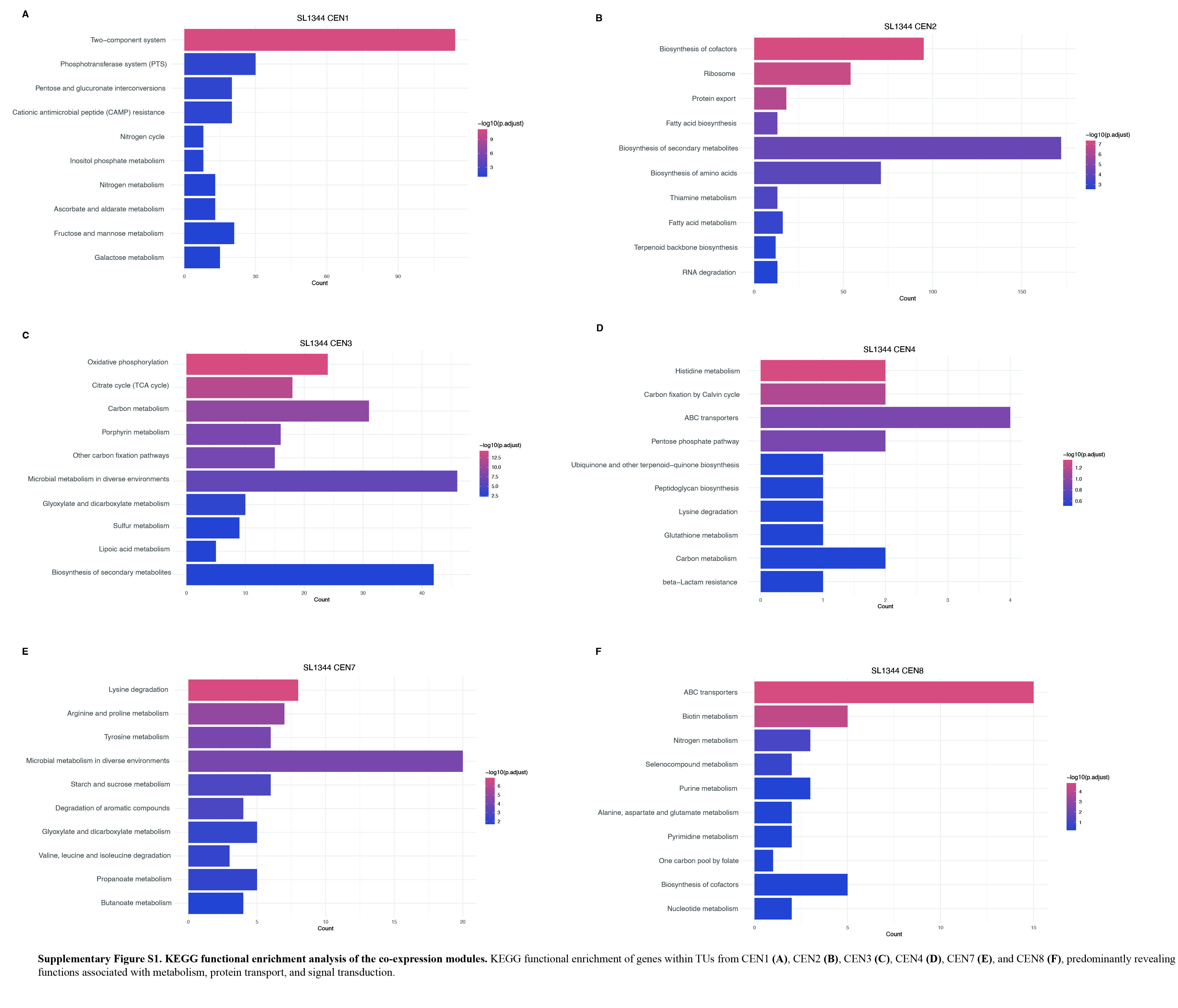

Supplement: Supplementary file 8 [file Image_1.jpeg]

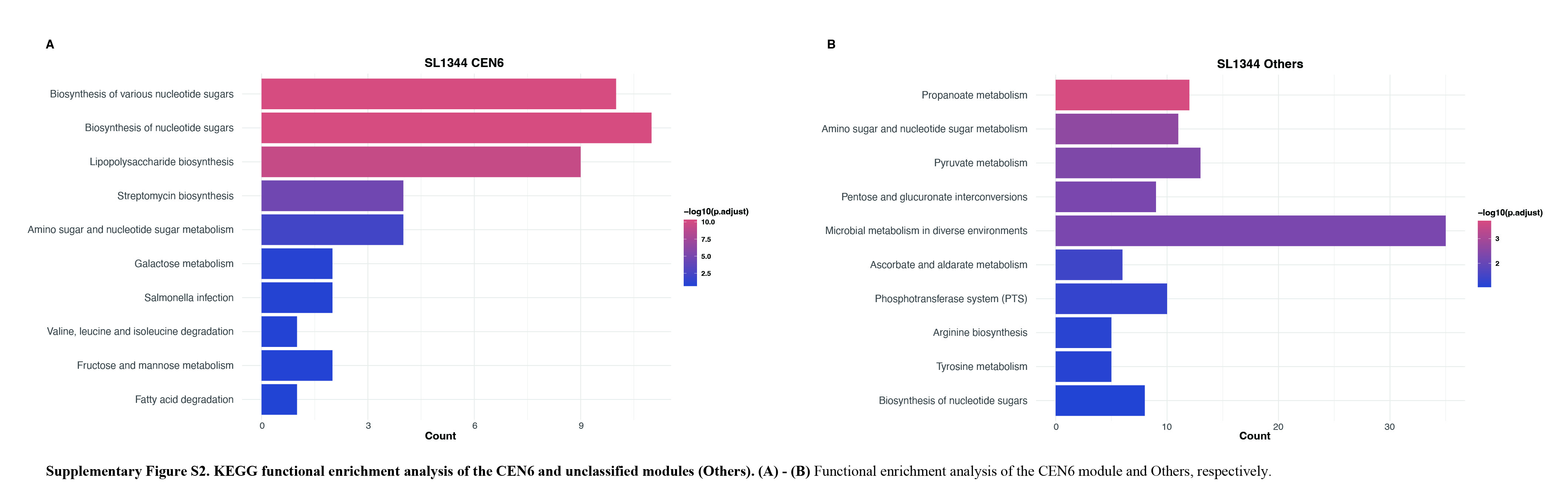

Supplement: Supplementary file 9 [file Image_2.jpeg]

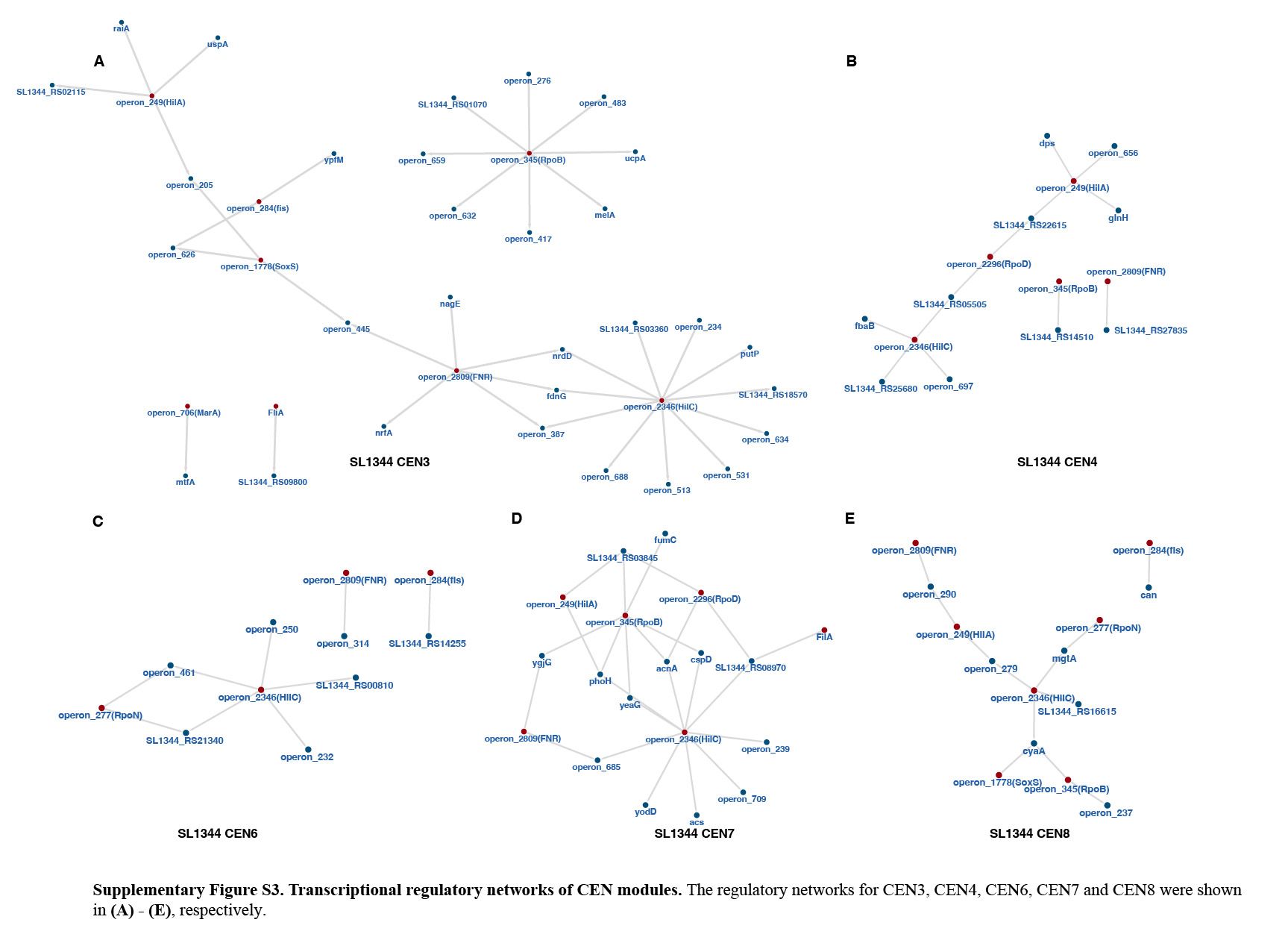

Supplement: Supplementary file 10 [file Image_3.jpeg]
